# Cardiac Troponin Serum Concentration Measurement Is Useful Not Only in the Diagnosis of Acute Cardiovascular Events

**DOI:** 10.3390/jpm14030230

**Published:** 2024-02-21

**Authors:** Grzegorz K. Jakubiak

**Affiliations:** Department and Clinic of Internal Medicine, Angiology, and Physical Medicine, Faculty of Medical Sciences in Zabrze, Medical University of Silesia, Batorego 15 St., 41-902 Bytom, Poland; grzegorz.k.jakubiak@gmail.com

**Keywords:** cardiac troponin, atherosclerosis, cardiovascular disease, subclinical cardiovascular dysfunction, myocardial injury

## Abstract

Cardiac troponin serum concentration is the primary marker used for the diagnosis of acute coronary syndrome. Moreover, the measurement of cardiac troponin concentration is important for risk stratification in patients with pulmonary embolism. The cardiac troponin level is also a general marker of myocardial damage, regardless of etiology. The purpose of this study is to conduct a literature review and present the most important information regarding the current state of knowledge on the cardiac troponin serum concentration in patients with chronic cardiovascular disease (CVD), as well as on the relationships between cardiac troponin serum concentration and features of subclinical cardiovascular dysfunction. According to research conducted to date, patients with CVDs, such as chronic coronary syndrome, chronic lower extremities’ ischemia, and cerebrovascular disease, are characterized by higher cardiac troponin concentrations than people without a CVD. Moreover, the literature data indicate that the concentration of cardiac troponin is correlated with markers of subclinical dysfunction of the cardiovascular system, such as the intima–media thickness, pulse wave velocity, ankle–brachial index, coronary artery calcium index (the Agatston score), and flow-mediated dilation. However, further research is needed in various patient subpopulations and in different clinical contexts.

## 1. Introduction

Cardiovascular diseases (CVDs) are one of the most important causes of morbidity and mortality worldwide [1], followed by cancers [2]. The main pathogenetic mechanisms leading to cardiovascular events include atherosclerosis [3], venous [4] and arterial thrombosis [5,6], and embolism [7,8,9,10]. Aneurysms [11,12,13], especially aortic aneurysms, and aortic dissection [14] also remain as important problems in the CVD field.

Methods for the diagnosis and treatment of CVDs have made significant progress in recent decades. At present, various diagnostic imaging methods (ultrasound, computed tomography, magnetic resonance imaging, nuclear medicine methods, invasive angiography) [15,16,17,18], as well as laboratory diagnostics, play an important role in the diagnosis of CVDs [19,20]. The development of therapeutic methods has been enabled by significant progress in the field of pharmacotherapy, as well as the development of surgical treatment methods (e.g., surgical revascularization within various vascular beds, embolectomy, and reconstructive procedures of the aorta and other arteries) [21,22,23,24,25,26], endovascular treatment techniques (e.g., balloon angioplasty, stenting, lithotripsy, local fibrinolysis, and mechanical thrombectomy) [27,28,29,30], and hybrid procedures [31].

Although approaches for the diagnosis and treatment of clinically overt CVD are currently at a very high level of development, an important problem is the search for methods that allow for the identification of patients with CVD at the subclinical or minimally symptomatic stage, in order to include such patients under intensified surveillance, providing more intensive control of risk factors as well as the use of invasive diagnostic and treatment techniques at the optimal moment. The tools used to assess subclinical dysfunction of the cardiovascular system include the ankle–brachial index (ABI) [32], intima–media thickness (IMT) [33], coronary artery calcium (CAC) score (or the Agatston score) [34], arterial stiffness assessment through pulse wave velocity (PWV) measurement [35], and assessment of endothelial function using the flow-mediated dilation (FMD) [36], among others. The mentioned parameters are briefly described in Table 1. The motivation for this literature review was to try to answer the question of whether, according to the currently available medical knowledge and the results of conducted research, a random measurement of cardiac troponin in a patient without symptoms of acute illness—including without suspicion of acute coronary syndrome—can be helpful in identifying patients with increased cardiovascular risk and cardiovascular dysfunction, as ascertained through the use of other methods.

The purpose of this paper is to present the results of a literature review focused on the possibility of using the measured cardiac troponin serum level outside the clinical context, in the case where an acute coronary syndrome is suspected in a patient.

## 2. Basics of Troponin Biochemistry and Its Release Mechanisms

An excellent review of the literature on the biochemistry of cardiac troponin, the mechanisms of its release from cardiomyocytes, and the clinical implications of these phenomena has been presented by Chaulin [37].

Troponin is a protein complex that occurs both in myocardial cells and skeletal muscle cells, which interacts with tropomyosin. It consists of three subunits: I, T, and C [38]. Troponin is the basic structural element responsible for the relationship between an increase in the calcium ion concentration in the muscle cell cytosol and activation of the contractile system of the cell [39]. Each subunit has precisely defined functions. Troponin T is responsible for the interaction with tropomyosin; troponin I is an inhibitory element that inhibits the hydrolysis of adenosine triphosphate in the absence of calcium ions and, thus, the interaction of actin and myosin in the diastolic phase; and troponin C is responsible for binding calcium ions [37]. It is worth noting that troponin C is not a marker specific to the heart muscle, due to the similarity of the amino acid sequence to troponin C present in skeletal muscles [40]. In the case of troponin T and troponin I, the difference in the amino acid sequence between the molecules present in myocardial cells and skeletal muscle cells allows them to be used in clinical practice as specific markers for myocardial injury [41,42].

The most important mechanisms responsible for the release of troponin from cardiomyocytes include renewal of the myocardium [43,44,45], apoptosis of cardiomyocytes [46,47], release through membrane vesicles [48], release of troponin proteolytic degradation fragments from cardiomyocytes [49,50], enhanced permeability of cardiomyocyte cell membranes [51,52], and small-scale myocardial cell necrosis [53,54].

## 3. Cardiac Troponin Blood Level in Acute Cardiovascular Events

According to the fourth universal definition of myocardial infarction, troponin I and troponin T are the most important markers of myocardial damage. If a change (i.e., increase or decrease) in the dynamics of the troponin concentration is observed, acute myocardial damage can be diagnosed. Myocardial damage may occur as a result of ischemia or for reasons unrelated to ischemia [55]. The basic mechanism leading to myocardial ischemia is rupture of the atherosclerotic plaque in the coronary arteries and the resulting thrombosis [56]. Other mechanisms of myocardial ischemia resulting from coronary artery pathology include coronary artery spasm [57] or dissection [58], as well as coronary embolism [59]. Ischemic myocardial damage may also result from disproportions between oxygen supply and demand due to conditions such as bradyarrhythmia [60], tachyarrhythmia [61], poorly controlled hypertension resulting in left ventricular hypertrophy [62], respiratory failure [63], hypotension [64], shock (different types, both cardiogenic and non-cardiogenic) [65,66,67], or severe anemia [68]. Moreover, important diseases in which elevated cardiac troponin levels are observed include heart failure [69,70,71], myocarditis [72,73], and chronic renal failure [74,75].

The cardiac troponin measurement results are important for the clinical evaluation of a patient diagnosed with pulmonary embolism. Elevated cardiac troponin levels indicate that this is a case of pulmonary embolism of at least intermediate risk [76,77]. In this case, increased cardiac troponin concentration indicates myocardial damage due to acute overload of the right ventricle. Careful clinical assessment of the patient and correct risk stratification in the case of a patient with pulmonary embolism are very important, as they allow the patient to be qualified for an appropriate treatment method among the available pharmacological treatments (anticoagulation, fibrinolysis) and invasive treatments (transcatheter procedures, embolectomy) [78,79].

Cardiac troponin levels may also increase in patients with acute aortic dissection [80], both Stanford A [81] and Stanford B types [82]. It has been shown that increased cardiac troponin levels in patients with aortic dissection have prognostic value and indicate an increased risk of death [83].

The most important causes leading to increased troponin serum concentration are summarized in Table 2.

## 4. Cardiac Troponin Serum Level in Chronic Cardiovascular Diseases

### 4.1. Chronic Coronary Syndrome

High-sensitivity cardiac troponin has been documented to be an independent predictor of obstructive coronary artery disease (CAD), assessed through computed tomography (CT) in patients during the diagnosis due to the suspicion of stable angina pectoris. The median troponin I concentration in patients with obstructive CAD was 1.9 (IQR: 1.3–3.1) ng/L while in patients without obstructive CAD, it was 1.2 ng/L (IQR: 0.8–1.9; *p* < 0.001) [89]. According to the results obtained in another study, an increased cardiac troponin T level is associated with a significantly increased rate of in-stent restenosis and de novo lesions, in comparison to patients with a serum troponin level within the normal range (28.6% vs. 10.3%; *p* < 0.05), considering patients with chronic coronary syndrome undergoing routine coronary angiography nine months after percutaneous coronary intervention (PCI) [90]. Another research team has presented data obtained from patients with suspicion of chronic coronary syndrome with a median troponin blood level of 7.8 ng/L (IQR: 4.8–11.6) whereas, in 18% of patients, the troponin blood level exceeded the upper reference limit (14 ng/L). In patients with an elevated troponin level, differences were found in comparison to patients with a normal troponin level, such as increased left ventricle mass (144 ± 40 g vs. 116 ± 34 g; *p* < 0.001), increased left ventricle volume (179 ± 80 mL vs. 158 ± 44 mL; *p* = 0.006), decreased left ventricle ejection fraction (LV EF) (59 ± 14% vs. 62 ± 11%; *p* = 0.006), decreased global longitudinal strain (14.1 ± 3.4% vs. 16.9 ± 3.2%; *p* < 0.001), more reversible perfusion defects (*p* = 0.001), and reversible wall motion abnormalities (*p* = 0.008) [91]. It has been documented that an increased cardiac troponin serum level at rest correlates with angiographic severity of CAD. No significant difference was found between the troponin isotypes (troponin T vs. troponin I) [92]. On the other hand, in patients at six months after acute coronary syndrome, mean troponin T is more often above the upper limit of the reference range than troponin I [93].

Interestingly, the increase in the troponin serum level after PCI in patients with stable angina is associated with the plaque structure, as assessed with CT. Features such as low plaque density, positive vessel remodeling, and spotty calcification are associated with significant post-PCI cardiac troponin serum elevation [94].

According to the results obtained in another study, in patients with suspected chronic coronary syndrome, increased troponin concentrations are associated with increased risk of incident atrial fibrillation [95].

A study involving 19,501 people from the general population showed that an increase in cardiac troponin I is associated with a significantly increased risk of myocardial infarction (HR: 1.18, 95% CI: 1.04–1.35) and coronary heart disease (HR: 1.19, 95% CI: 1.11–1.28); however, a similar effect was not found for cardiac troponin T. Similarly, an increase in troponin concentration was associated with a significantly increased risk of death from cardiovascular causes (in the case of both troponin T and troponin I) [96]. Another study conducted in the general population has shown that the measurement of ultrasensitive troponin I may be a useful marker for increased risk of coronary heart disease [97].

### 4.2. Chronic Lower Extremity Ischemia

It was found that detectable cardiac troponin (≥5 ng/L) is associated with significantly higher risk of symptomatic peripheral arterial disease, that is, stage IIa or IIb in the Fontaine scale (OR: 1.84, 95% CI: 1.05–3.21; *p* = 0.03). Moreover, cardiac troponin concentration above or equal to 14 ng/L was associated with a significantly higher risk of death (HR: 5.06, 95% CI: 2.23–10.85; *p* < 0.001) and risk of cardiovascular events (HR: 3.15, 95% CI: 1.26–7.89; *p* = 0.01) when considering a median follow-up period of seven years [98]. Similarly, according to the results obtained in another study, troponin concentration above or equal to 14 ng/L in patients with intermittent claudication without symptoms of heart disease was associated with a significantly increased risk of cardiac death, myocardial infarction, or urgent revascularization during follow-up [99]. It was found that patients with chronic limb-threatening ischemia (CLTI)—previously named critical limb ischemia (CLI)—present higher severity of CAD and are characterized by higher troponin serum concentration than patients with intermittent claudication (152 ± 186 ng/L vs. 46 ± 91 ng/L; *p* < 0.0001) [100]. Similar conclusions were obtained in the systematic review and meta-analysis of eight observational studies. According to the results of this study, elevated troponin concentration was observed in 5.3% (range: 4.4%–8.7%) of patients with intermittent claudication and in 62.6% (range: 33.6%–85%) of patients with CLTI. An elevated troponin level was significantly associated with an increased risk of all-cause mortality (HR: 2.85, 95% CI: 2.28–3.57) and risk of major adverse cardiovascular events (HR: 2.58, 95% CI: 2.04–3.26) [101].

Patients with chronic lower extremity ischemia—especially those with CLTI—often have clinically obvious and advanced atherosclerosis in other vascular beds, including coronary arteries. The features of myocardial damage in this patient population can, therefore, be interpreted as a manifestation of the overall poor condition of the cardiovascular system [102].

### 4.3. Cerebrovascular Disease

An increased baseline troponin level was associated with significantly higher risk of incidence of cerebrovascular events (HR: 4.57, 95% CI: 1.08–19.22; *p* < 0.05) in memory-clinic patients aged 74 ± 10 years [103]. Patients after ischemic stroke with an elevated troponin serum level had significantly higher risk for the occurrence of another stroke than subjects with a normal troponin serum level (adjusted HR: 1.73, 95% CI: 1.07–2.78; *p* = 0.02), as well as significantly increased risk of other cardiovascular events such as coronary revascularization and heart failure (median follow-up: 22.5 months, IQR: 5.0–38.8) [104].

An elevated serum troponin I level was shown to be associated with an increased risk of cardiovascular and cerebrovascular events in patients with atrial fibrillation and concomitant heart failure with a preserved ejection fraction (median follow-up: 24.2 months, IQR: 7.5–38.6) [105].

It has been documented that the HEART score, which takes into consideration history, ECG, age, risk factors, and troponin level, predicts recurrent cerebral ischemic event risk as well as mortality within 30 days and 1 year in patients with acute ischemic stroke [106].

According to the results of the previously mentioned study conducted on the general population, an increase in troponin concentration was associated with a significant increase in the risk of stroke during the observation period. This relationship was statistically significant both for troponin I (HR: 1.39; 95% CI: 1.21–1.6) and troponin T (HR: 1.22; 95% CI: 1.04–1.43) [96].

## 5. Cardiac Troponin Serum Level and Subclinical Cardiovascular Dysfunction

### 5.1. Intima–Media Thickness

The troponin serum level may be considered as a useful tool for the prediction of subclinical atherosclerosis. According to a study, which included 1745 women and 1666 men without diagnosed CAD, the troponin serum level was significantly correlated with carotid IMT (*r* = 0.14, *p* < 0.001) [107]. Another research team has presented the results of an analysis of 4139 subjects from the population-based Gutenberg Health Study, based on which a significant association was found between the cardiac troponin I serum concentration and the carotid IMT value (*β* = 0.061, *p* < 0.001 in men; *β* = 0.047, *p* = 0.013 in women) [108].

Another publication has documented a significant correlation between the cardiac troponin serum level and carotid IMT in a group of 120 pre-dialysis adults with chronic kidney disease (*r* = 0.419, *p* < 0.0001). Similarly, carotid IMT also correlated with other cardiovascular biomarkers such as the N-terminal pro-B-type natriuretic peptide (NT-proBNP), high-sensitivity C-reactive protein (hs-CRP), and albuminuria; however, when compared with troponin, the correlation was stronger only for NT-proBNP (*r* = 0.575, *p* < 0.0001) [109]. Similarly, a study conducted on a group of hemodialysis patients showed that patients with elevated cardiac troponin serum concentration above the cut-off point for diagnosing myocardial damage (>0.1 µg/L) were characterized by a significantly higher carotid IMT value, compared to those with troponin concentration below the cut-off point (0.85 vs. 0.70 mm; *p* < 0.011) [110]. Another study involving 52 people with end-stage renal disease (25 undergoing hemodialysis and 27 undergoing peritoneal dialysis) also reported a significant correlation between cardiac troponin concentration and the carotid IMT value (*r* = 0.35; *p* = 0.003) [111]. A study involving 72 non-diabetic hemodialyzed patients with a mean age of 34.5 ± 10.6 years also showed a significant correlation between cardiac troponin concentration and the carotid IMT value [112]. In a study performed on a group of 37 peritoneal dialysis patients, the troponin T serum level significantly correlated with carotid IMT (*r* = 0.747, *p* < 0.001). Furthermore, in their multivariate analysis, troponin remained a significant independent predictor of carotid IMT (*β* = 4.446, *p* < 0.001) [113]. Chronic kidney disease is a strong cardiovascular risk factor, as CVDs are the leading cause of death in this population [114].

Cardiac troponin serum concentration correlated significantly with carotid IMT in patients with rheumatoid arthritis (*r* = 0.337, *p* = 0.002). Moreover, median troponin serum concentration was significantly higher in patients with rheumatoid arthritis, when compared with controls (0.02, IQR: 0.10 vs. 0.0, IQR: 0.01; *p* = 0.001), as well as median carotid IMT (0.9 mm, IQR: 0.2 vs. 0.7 mm, IQR: 0.1; *p* = 0.001) [115].

Another research team has presented the results of a study involving 226 people with type 2 diabetes. Using a linear regression model, it was shown that cardiac troponin concentration was significantly associated with carotid IMT in the total cohort (*ß_st_* = 0.27, *p* < 0.0001), although increased troponin serum concentration was observed only in nine patients (4%). A similar effect was found among the men, when analyzed separately (*ß_st_* = 0.34; *p* < 0.0001), but not among the women (*ß_st_* = 0.05; *p* = 0.6142). It is worth noting that, in the study population, the percentage of patients diagnosed with atherosclerotic CVD was significantly higher among men than among women (41% vs. 18%; *p* < 0.0001) [116]. Another study has shown that, in a group of patients with type 2 diabetes, those with an above-average accumulation of advanced glycation products (assessed through skin autofluorescence) were characterized by both a higher cardiac troponin serum concentration (*p* < 0.0001) and a higher carotid IMT value (*p* = 0.017) [117].

### 5.2. Pulse Wave Velocity

In the abovementioned research performed by Caliskan et al., among 37 peritoneal dialysis patients, the troponin T serum level was also significantly correlated with PWV (*r* = 0.431, *p* = 0.035) [113]. According to the results obtained in a study in which data from 176 hemodialysis patients were analyzed, troponin T concentration was significantly correlated with brachial–ankle PWV (*r* = 0.5, *p* < 0.01). Moreover, the relationship remained significant when using the multivariable-adjusted linear regression model, taking into account age, gender, smoking, blood pressure, and comorbidities, among others [118].

It has been found that, among women diagnosed with systemic lupus erythematosus, the percentage of patients with increased PWV was significantly higher among patients with a detectable troponin I level than among patients with an undetectable troponin I level (38% vs. 12%; *p* = 0.024) [119].

The troponin T serum level was significantly correlated with brachial–ankle PWV (*r* = 0.316, *p* < 0.0001) in patients with essential hypertension [120]. In patients with type 2 diabetes, the troponin I serum level was correlated with brachial–ankle PWV (*r* = 0.34, *p* < 0.01), as well as serum creatinine (*r* = 0.26, *p* = 0.01) and systolic blood pressure (*r* = 0.31, *p* < 0.01). Interestingly, linear logistic regression showed that only brachial–ankle PWV was independently associated with the troponin serum level (*β* = 0.25, *p* = 0.04) [121].

In a study performed on 74 patients, it was found that, twelve months after ST-elevation myocardial infarction, the troponin T serum level correlated significantly with PWV (*r* = 0.435, *p* < 0.001), although the troponin level was within the normal range for the majority of participants (median: 6.4 ng/L, IQR: 5.0–8.6) [122]. Ki et al. have shown that brachial–ankle PWV has a greater prognostic value in assessing the risk of cardiac death after PCI in the subpopulation of patients with a maximum troponin T serum concentration of at least 0.1 ng/mL, compared to patients with a lower maximum troponin serum concentration during hospitalization. The average follow-up period was 25.8 months [123].

Carotid–femoral PWV has been shown to be associated with minimally elevated troponin T serum levels in the elderly, in terms of detectability (OR: 1.84, 95% CI: 1.06–3.17; *p* = 0.028) as well as its increased level (OR: 2.34, 95% CI: 1.03–5.30; *p* = 0.042), when adjusted for age, sex, hypertension, diabetes, current smoking, body mass index, heart rate, blood pressure, renal function, serum lipid profile, fasting glucose, and hs-CRP. On the other hand, carotid–radial PWV was associated with the troponin T serum level only in the univariate analysis, with loss of the significance after adjustment for the abovementioned factors [124]. No significant association was found between PWV and a minimally elevated troponin level in a middle-aged male worksite cohort (2374 male employees of a company with a mean age of 46 ± 9 years) [125].

### 5.3. Ankle–Brachial Index

It has been documented that an increased serum troponin level is independently associated with increased risk for the occurrence of peripheral arterial disease, understood as a decrease in the ABI [126]. A significant correlation has been reported between the ABI (taking into consideration subjects with ABI not exceeding 1.4) and troponin T serum level in patients with type 2 diabetes (*r* = −0.1; *p* < 0.01). A total of 1066 patients aged 60–75 years participated in the study, with part of the study group consisting of people previously diagnosed with CVD [127].

Data from people aged 40 years or older without overt CVD participating in the NHANES study from 1999 to 2014 have been analyzed and it was shown that, among people with a reduced ABI value (<0.9), 73.9 ± 3.5% presented a serum troponin T concentration > 6 ng/L, while 32.3 ± 3.7% had a serum troponin I concentration ≥ 6 ng/L for men and ≥4 ng/L for women [128].

In a study conducted on a cohort of 10,897 people who did not have diagnosed CVD at baseline, it was found that the coexistence of increased serum troponin T concentration (≥14 ng/L) and a decreased ABI value (<0.9) is associated with a higher risk of developing atherosclerotic CVDs within an average observation period of 13.6 years than in the case of taking each of these factors into consideration separately [129].

In patients with cerebral artery atherosclerosis, positive troponin (OR: 2.76, 95% CI: 1.69–4.53; *p* < 0.001) as well as low ABI (OR: 3.25, 95% CI: 1.21–8.73; *p* = 0.019) were found to be predictors of asymptomatic angiographically significant CAD [130]. Asymptomatically decreased ABI was shown to be associated with an increased risk of perioperative myocardial damage, as assessed through troponin level measurements in vascular surgery patients [131]. Similarly, in another study, decreased ABI was shown to be associated with an increased risk for isolated troponin elevation after non-cardiac surgery (OR: 7.4, 95% CI: 2.2–25.0; *p* = 0.001) [132].

### 5.4. Calcium Score

Coronary artery calcification, assessed through chest computed tomography, has been shown to be associated with the features of myocardial injury in COVID-19 survivors. The cardiac troponin serum level was significantly higher in patients with coronary artery calcification than those without coronary calcification (19.3 ± 18.0 ng/L vs. 10.3 ± 10.8 ng/L; *p* < 0.01) [133]. In patients at least fifty years old with well-controlled human immunodeficiency virus infection and without evidence of coronary artery disease, it has been found that troponin T and troponin I correlate significantly with the Agatston score (*r* = 0.27 and *r* = 0.28, respectively; *p* < 0.001) [134].

In a study performed in a group of very elderly patients, it was found that the troponin T serum level increased with a higher Agatston score [135].

Korley et al. have performed an interesting pilot study, in which they documented that a low high-sensitivity troponin I serum level coexisting with zero CAC may allow for the avoidance of further diagnostics in the coronary arteries [136].

### 5.5. Flow-Mediated Dilation

In a study conducted on 2952 Japanese people from the general population, it has been shown that those with FMD < 5% had a significantly higher median cardiac troponin concentration than those with FMD ≥ 5% (6 ng/L vs. 5 ng/L; *p* < 0.001) [137]. In another study, performed on 4139 subjects from the general population, only a borderline correlation between the troponin I level and FMD value was observed (*p* = 0.05 for women and *p* = 0.018 for men) [108]. It is worth noting that the FMD is a technically difficult test, which requires extensive experience on the part of the operator and the center where it is performed; therefore, there are possible discrepancies between the results of various studies available in the literature.

A research team from the United States has conducted a study on a group of 187 people undergoing vascular surgery, in order to investigate the usefulness of assessing endothelial function using the FMD method to assess the risk of postoperative cardiovascular events. It was shown that, among patients whose FMD value does not exceed 8.1%, there is a significantly more frequent postoperative isolated increase in cardiac troponin concentration (*p* < 0.05) [138].

In a prospective observational study involving patients hospitalized in an intensive care unit due to COVID-19, it has been documented that the troponin serum level correlated significantly with endothelial function assessment through FMD (*r* = −0.45, *p* < 0.001). Moreover, FMD also correlated with higher lung parenchymal involvement. The troponin level was significantly higher in non-survivors than in those discharged alive (194.79  ±  283.25 ng/L vs. 18.41  ±  20.8 ng/L; *p* < 0.001) [139]. Perhaps the observed association between the troponin level and FMD is an effect of the severe general clinical state of study participants in respiratory failure. Similarly, in another study performed on a group of 72 patients in the acute phase of COVID-19 (37% admitted to the ICU), a significant association between the FMD and troponin I level was observed [140]. Additionally, in patients in the acute phase of takotsubo cardiomyopathy, a significant correlation was observed between the troponin I peak and FMD value (*r* = −0.7645, *p* < 0.001) [141].

## 6. Conclusions

Cardiac troponin is the basic marker measured in patients suspected of acute coronary syndrome and also plays an important role in patients with diagnosed pulmonary embolism, in terms of stratifying the risk of hemodynamic instability. Cardiac troponin levels may also increase in patients with aortic dissection, thus possessing prognostic significance in this population. Serum cardiac troponin concentration is also a general marker of myocardial damage, regardless of etiology, which may occur in the course of many diseases, including primary non-cardiogenic diseases.

According to the results of the conducted literature review, cardiac troponin concentration may also increase in patients with chronic atherosclerotic CVD, such as chronic coronary syndrome, chronic lower extremities’ ischemia, or cerebrovascular disease. Cardiac troponin concentration has certain prognostic value in these patients.

Moreover, a number of publications have shown that the concentration of cardiac troponin correlates with some parameters indicating subclinical dysfunction of the cardiovascular system, although the results of the studies conducted so far are not fully clear. This literature review described the relationships between cardiac troponin concentration and parameters such as the IMT, ABI, CAC, PWV, and FMD. It should be noted that the results of the mentioned studies depend largely on the methodology used (e.g., the section on which the pulse wave velocity was measured or the special equipment used to assess endothelial function using the FMD method); hence, there may be discrepancies between the results of various studies. Moreover, the studies cited in this literature review mostly considered various specific subpopulations of patients, such as patients with chronic renal failure, patients with selected rheumatological diseases, and patients with type 2 diabetes, while only some studies focused on people from the general population or without diagnosed CVD.

Further research should be focused primarily on people from the general population, in order to verify the hypothesis that cardiac troponin concentration correlates with markers of subclinical cardiovascular dysfunction and that such a correlation translates into the risk of developing overt CVD and the occurrence of cardiovascular events. It would also be worth examining the relationship between the cardiac troponin concentration and additional parameters, such as the toe–brachial index (TBI) and pulse wave analysis parameters. It is worth noting that the importance of cardiac troponin as a potential marker for cardiovascular screening has already been discussed in the literature [142].

It is also worth examining the differences and similarities between troponin T and troponin I in more detail, in terms of their relationships with the presence of chronic CVD and the severity of subclinical cardiovascular dysfunction. In most of the cited studies, only one of these parameters was measured. The differences in the methodologies of many of the cited studies are so significant that it is difficult to compare different studies that measured troponin T or troponin I.

## Figures and Tables

**Table 1:** Description of the selected parameters used for determination of subclinical cardiovascular dysfunction.

Ankle–brachial index (ABI)	The ratio of blood pressure at the ankle to blood pressure at the arm level [32].
Intima–media thickness (IMT)	Total thickness of the intima and media measurement using ultrasound in B presentation, most often performed in the common carotid artery [33].
Coronary artery calcium (CAC) score	Assessment of coronary artery calcium using computed tomography (CT) [34].
Pulse wave velocity (PWV)	Assessment of arterial stiffness through measuring the speed of information propagation along the arterial wall [35].
Flow-mediated dilation (FMD)	Ultrasound assessment of the change in the diameter of the brachial artery under the influence of reactive hyperemia [36].

**Table 2:** The most important causes leading to increased troponin serum concentration.

Cause of Increased Troponin Serum Concentration		Reference
Myocardial ischemia due to pathology in the coronary arteries	Destabilization of atherosclerotic plaque	[56]
	Coronary artery spasm	[57]
	Coronary artery dissection	[58]
	Coronary embolism	[59]
Myocardial ischemia due to other pathologies than coronary arteries’ dysfunction	Bradyarrhythmia, tachyarrhythmia	[60,61]
	Poorly controlled hypertension resulting in left ventricular hypertrophy	[62]
	Respiratory failure	[63]
	Hypotension, shock (different types, both cardiogenic and non-cardiogenic)	[64,65,66,67]
	Severe anemia	[68]
Other pathologies	Heart failure	[69,70,71]
	Myocarditis	[72,73]
	Chronic renal failure	[74,75]
	Pulmonary embolism (of at least intermediate risk)	[76,77]
	Aortic dissection	[80,81,82,83]
Other causes	Physical activity	[84,85,86]
	Heart trauma	[87]
	Drug-induced cardiotoxicity (e.g., anthracyclines)	[88]

## Data Availability

Not applicable.
